# Early Complication Analysis of Dynamic Intraligamentary Stabilization versus Anterior Cruciate Ligament Reconstruction

**DOI:** 10.3390/jpm13071022

**Published:** 2023-06-21

**Authors:** Frank Endreß, Reinhard Hörner, Wolfgang Hauth, Jens Anders, Roland Biber

**Affiliations:** 1 Kliniken Dr. Erler gGmbH, Kontumazgarten 4-19, 90429 Nürnberg, Germany; f.endress@erler-klinik.de (F.E.);; 2 Friedrich-Alexander-University Erlangen-Nürnberg (FAU), Schloßplatz 4, 91054 Erlangen, Germany; 3 Paracelsus Medical University (PMU), Prof.-Ernst-Nathan-Str. 1, 90419 Nürnberg, Germany

**Keywords:** anterior cruciate ligament, reconstruction, dynamic intraligamentary stabilization, range-of-motion

## Abstract

Purpose: Both dynamic intraligamentary stabilization (DIS) and reconstruction (RECO) are common treatment methods for anterior cruciate ligament (ACL) rupture. We report short term outcomes after DIS (Ligamys, Mathys, Bettlach, Switzerland) and RECO using semitendinosus tendon. We compared postoperative complications, deficits of range-of-motion (ROM), and revision rates between the two treatment options. Methods: A total of 690 patients (437 male, 253 female), after either DIS or RECO, were included. Of these, 147 patients (21%) received DIS and 543 (79%) underwent RECO. Follow-up examination focused on clinical examination, complications and revision rates. Anteroposterior instability and ROM deficits were analyzed in order to evaluate our policy of early intervention for all cases of ROM restrictions. Results: Relevant ROM restrictions occurred at a significantly higher rate after DIS than after RECO (4.8% vs. 1.3%; *p* = 0.008). Flexion was more restricted after DIS than RECO (110° vs. 124°, *p* < 0.001). Extension deficits also occurred more frequently after DIS compared to RECO (49.7% vs. 24.5%; *p* < 0.001). Total revision surgery rate was 9.1%, with patients after DIS being significantly more frequently affected (20.4% vs. 6.1%; *p* < 0.001). Conclusions: Our findings indicate a significantly higher risk for ROM restriction after DIS compared to RECO, resulting in a significantly higher revision rate.

## 1. Introduction

Knee injuries account for a high number of sports-related surgeries [1,2,3,4,5,6,7]. Anterior cruciate ligament (ACL) rupture is one of the most common sports injuries. The incidence varies from 36.9–78/100,000 inhabitants worldwide [4,6,8,9]. Males have a higher risk of sustaining an ACL injury than females [8,9,10]. Up to 65% of ACL injuries result in surgery [9]. In Germany alone, more than 40,000 anterior cruciate ligament surgeries are performed annually, demonstrating, together with other reports, the relevance of ACL ruptures in today’s orthopedics [4,6,8,9,10]. 

Injuries of the ACL rarely occur in isolation. The impact of concomitant injuries, including other ligament sprains, meniscal tears, articular cartilage injuries and bone bruises, complicate the treatment and potential outcomes of ACL tears [8,11]. Reported meniscal injuries associated with acute ACL rupture range from 15% to more than 50% [11,12,13] and are among the most common injuries requiring additional surgery.

According to the guidelines of the German Society for Trauma Surgery (DGU) [8], conservative treatment of ACL tears is recommended only for elderly patients or patients with reduced activity requirements and no persistent symptoms of instability. However, there is no general age limit for surgical ACL reconstruction [8,14]. Elderly and physically active patients who are not satisfied with conservative treatment may also be considered for a knee stabilizing procedure provided they do not suffer from arthritic ACL-deficient knees [8,14].

Cruciate ligament surgery is subject to the constant development of anatomical and biomechanical knowledge, and consequently, of both surgical techniques and postoperative treatment. Today’s gold standard for ACL surgery is ACL reconstruction (RECO), primarily developed in 1966 by H. Brückner, who proposed harvesting the middle third of the patellar tendon [15,16,17]. The first publication describing a technique using hamstring tendon grafts was by B. Lipscomb in 1982 [18]. In 2022, Liukkonen et.al. demonstrated that RECO is a reliable procedure with overall low historical revision rates [19]. Between 1969 and 2018, there was an overall revision rate of 3.14%, of which 2.71% related to hamstring autografts and 2.38% related to bone–patellar tendon–bone (BPTB) autografts [19].

In medical history, the procedure of ACL replacement surgery has evolved as a result of failures after primary ACL suturing [20]. The importance of repair after ACL ruptures due to the inability of spontaneous healing was published by Ivar Palmer in 1938, in which he presented a technique for primary suturing of the acutely torn anterior cruciate ligament [21]. However, the results of early repair techniques were not convincing, and overall failure and recurrence rates were very high [17,22,23,24,25]. Nevertheless, interest in preserving the anterior cruciate ligament has continued over the years. Several studies have shown that the cruciate ligaments, which have a proportion of mechanoreceptor nerve fibers in their structure (1 to 2 percent nerve fibers by volume), play an important role in proprioception of the knee joint and thus the lower extremity [20,26,27].

Anatomical and histological examinations of the knee joint have also demonstrated that the anterior cruciate ligament has healing potential [20,28,29,30,31,32,33]. The ability of the ACL to heal eventually led to the development of “dynamic intraligamentary stabilization” (DIS) of the anterior cruciate ligament, Ligamys, by Eggli et. al. and Kohl et al. [17,20,30,34,35,36]. In a comparative study from 2008, it was demonstrated that, in contrast to non-augmented ACL suture repair and static tape augmentation, only dynamic augmentation (Ligamys) resulted in restoration of anterior tibial translation values similar to the ACL-intact knee [37].

The biomechanical objective of Ligamys restoration is to provide temporary stabilization to the knee joint using a dynamic spring system, essentially serving as an artificial replacement for the ACL [38]. In each position, a defined spring force of 60–80 Newtons pulls the tibial head dorsally [38]. By means of this technique, it is ensured that the knee is locked in a fixed posterior translation at any degree of flexion, keeping the two ligamentous stumps in the closest possible position at all times [17]. Thereby, the reattached ACL maintains the necessary contact to be able to scar stably [17,38,39,40]. During the first six postoperative months, the implanted polyethylene thread “slips out” of the spring clamping mechanism in a controlled manner. The original cruciate ligament increasingly takes over the stabilization within 6 months. After this time, the implant suture no longer has tension in the spring clamping mechanism [17,30,38,40]. 

In our clinic, we use either the conventional technique of anterior cruciate ligament replacement with transtibial femoral drilling or the Ligamys technique as surgical treatment of anterior cruciate ligament injuries, depending on the indication. According to the current standard, indications for Ligamys treatment are fresh proximal cruciate ligament ruptures that are not older than 21 days. Contraindications are currently ACL ruptures older than 21 days, acute or chronic infections (local or systemic), arthrofibrosis, severe malposition of the knee joint, severe muscle, nerve or vascular diseases, hypersensitivity to the materials used (e.g., cobalt, chromium, nickel, etc.), insufficient bone substance and poor bone quality, which could endanger a stable anchoring of the implant. A relative contraindication is for children and adolescents with open epiphyseal gap [20,40]. According to several studies, another factor acting as a potential determinant of poor outcome after ACL repair using the DIS technique, was a high pre-injury sports activity level [41,42,43].

The aim of this study is to thoroughly evaluate conspicuous clinical findings and early complications during the follow-up period, in particular signs of infection, ROM deficits, incipient arthrofibrosis and the resulting revision rates after ACL rupture, comparing DIS and RECO. The knowledge gained from the study will be used in the future to develop optimized treatment strategies tailored to the individual patient, taking into account the patient’s individual goals. In addition, any early complications should be detected in time and counteracted in a guideline- and evidence-based manner.

## 2. Patients and Methods

For this single-center prospective study, each patient treated either by ACL dynamic intraligamentary stabilization (DIS) or by ACL reconstruction (RECO) was invited for follow-up 6 weeks and 3 months postoperatively. All patients attending at least one follow-up examination were included. Data were collected from 1148 consecutive follow-up examinations from 690 patients between the years from 2012 until 2022.

**Figure 1:**
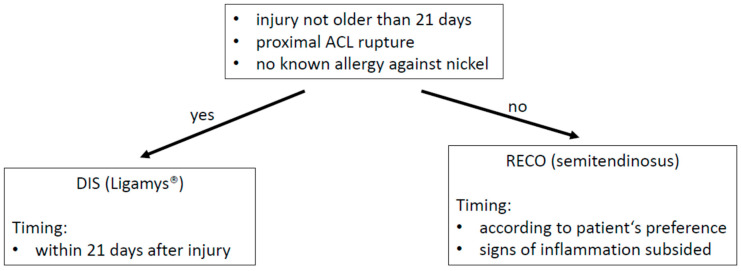
Treatment algorithm.

Whenever DIS indication criteria were fulfilled, we recommended DIS in order to preserve the patient’s natural ACL. Otherwise, we advocated for RECO as soon as all signs of inflammation had subsided and range-of-motion (ROM) had returned to normal (see Figure 1 for our treatment algorithm). 

In total, 147 patients (21%) received DIS and 543 patients (79%) received ACL RECO. DIS was performed using the Ligamys method by Mathys Ltd. (Bettlach, Switzerland) according to manufacturer’s instructions. For RECO, we used a transtibial technique (POSITION ACL, B. Braun Aesculap, Tuttlingen, Germany) with semitendinosus tendon as autograft [44]. The Ultrabutton Adjustable Fixation System from Smith&Nephew (London, UK) served as a suture plate for femoral implant anchoring and the Königsee Button (Allendorf, Germany) served as the suture disc for the tibial anchor. Drainages were routinely used in RECO, but not in DIS. 

All surgeries, irrespective of the method used, were performed with the patient positioned in supine position and under general anesthesia. The knee joint was stored using a knee holder with, intraoperatively, 130-degree flexion ability. A tourniquet was inflated to 300–350 mm Hg. The arthroscopy portals for both surgical techniques are the same, with the exception that a larger incision is recommended for the anteromedial portal in the Ligamys treatment [40]. In both DIS and RECO, concomitant injuries were treated prior to the ACL treatment procedure. For both methods, we used devices for implant tension control. In DIS procedures, a tensiometer and a torque wrench were used to pretension the Ligamys tape to the maximum in 90° flexion. In full extension, the clamping cone was screwed in with a pretension of 60 to 80 Newtons. In RECO procedures, tibial anchorage was established using an Ultrabutton (Smith & Nephew). Fixation was achieved by knotting with a knot aid, and under tension, in the near-extension position. Residual laxity was checked and (if present) eliminated by rotating the Ultrabutton with the aid of the twister. We checked that the Ultrabutton resisted lift-off during application of at least 75 Newton meters. 

Postoperative mobilization was performed during the inpatient stay according to the respective in-house post-treatment protocols. The protocols were based on the post-treatment recommendations of the manufacturers and were handed out to the patients upon discharge [45]. These included strict advice to attend continuous physiotherapy after discharge. Scheduled postoperative follow-up visits at our clinic are recommended after 6 and 12 weeks. 

Our collective consisted of 437 males and 253 females (63.3% vs. 36.7%). The mean age was 32.0 years (SD 11.1 years; range 12 to 73 years). A total of 690 surgeries were performed, including 147 DIS and 543 RECO. Postoperative treatment protocols after DIS and RECO differed only slightly; both protocols were based on the post-treatment recommendations of the respective transplant manufacturers and were handed out to the patients upon discharge. Both protocols included the advice for immediate active physiotherapy without ROM restriction; however, such restrictions could have applied in cases of combined procedures (e.g., simultaneous meniscus surgery). 

Scheduled postoperative follow-up visits at our clinic were offered to every ACL patient and were recommended after 6 and 12 weeks. These examinations aimed at early identification of patients with ROM deficits or incipient arthrofibrosis, which we regard as indication for early arthroscopic adhesiolysis. 

Parameters evaluated during initial treatment and follow-up included:

- age and gender;
- kind of index surgery including combined procedures;
- ROM (measurement by standard goniometer);
- anteroposterior instability (measured by rolimeter) compared to the contralateral knee joint;
- clinical presence of classical inflammation signs: effusion, knee swelling and rubor;
- mean circumferences of the thigh, knee, and lower leg;
- rehabilitation activities / physiotherapy;
- revision surgery rates and procedures;
- subjective satisfaction of patient and surgeon on a scale of 1 (best) to 6 (worst).

Statistical analysis calculated both descriptive and inferential measures. Mean values and standard deviations were calculated for continuous variables, and frequency counts and percentages were obtained for the discrete variables. All confidence intervals (designated “CI”) are 95% confidence intervals. The χ^2^ test was used for cross tabulation analysis when all expected cell frequencies were five or greater; Fisher’s exact test was used otherwise. For continuous variables, the two-sample independent *t*-test was used. Nonparametric correlations were evaluated using Spearman’s ρ. All statistical analyses were performed with IBM SPSS Statistics Version 19. 

## 3. Results 

We report on the outcomes of a total of 690 cases, consisting of 147 DIS (21%) and 543 RECO (79%). Mean operation time did not relevantly differ between these two methods (55 min versus 53 min). 

A total of 437 of all patients were male (63.3%). This proportion turned out to be different between the DIS and RECO subgroups: 65.7% of ACL-reconstruction patients ware male, whereas this proportion was only 54.4% in the DIS group (difference significant on χ^2^ test, *p* = 0.01). 

**Figure 2:**
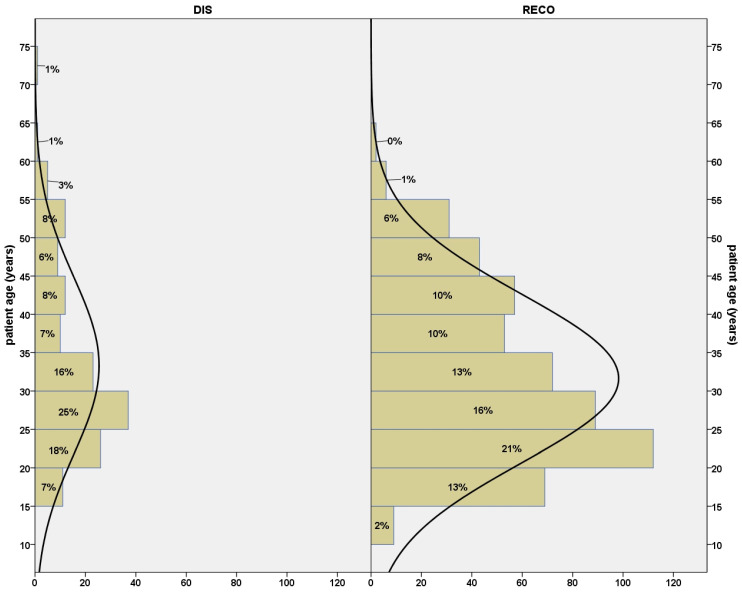
Age distributions of DIS and RECO patients did not differ significantly.

The patient ages ranged from 12 years to 73 years; the mean age was 32.0 years (SD 11.1 years). The mean patient age did not significantly differ between DIS and RECO (33.3 years versus 31.6 years; *p* = 0.1), as is shown in Figure 2. On average, female patients were slightly older than male patients (34.0 years versus 30.9 years; *p* < 0.001). Regarding the subgroups, this was true only for RECO patients (34.4 years versus 30.2 years; *p* < 0.001), whereas female DIS patients tended to be younger (32.8 years versus 33.8 years; not significant).

Index operation was combined with medial meniscus surgery in 185 cases (resection: 14.9%; suture: 11.9%), lateral meniscus surgery in 147 cases (resection: 14.4%; suture: 7.0%), medial ligament injury treatment in 14 cases (conservative: 0.9%; operative: 1.2%), cartilage surgery in 10 cases (1.5%) and PCL treatment in 4 cases (conservative: 0.1%; reconstruction: 0.4%). DIS was significantly more often accompanied by meniscal surgery than RECO (50.3% vs. 40.1%; *p* = 0.026). Details are provided in Table 1. 

### Follow-Up

The mean duration until first follow-up was 53 days. Between hospital discharge and follow-up, 15 RECO patients had started or completed an inpatient rehabilitation program (2.2%). For outpatient rehabilitation programs these rates were higher (35.2% in the DIS and 41.6% in the RECO group). A total of 31.7% of DIS patients and 41.4% of RECO patients declared that they had not received any physiotherapy or rehab at all after being discharged from our hospital. 

We did not detect any cases of infection or implant failure on first follow-up. When evaluating satisfaction on a subjective basis, using a scale of 1 (best) to 6 (worst), patient and surgeon perfectly agreed on a mean rating of 2.3 after RECO. DIS patients’ mean rating was 2.6, as opposed to the surgeons’ mean rating of 3.1. 

**Figure 3:**
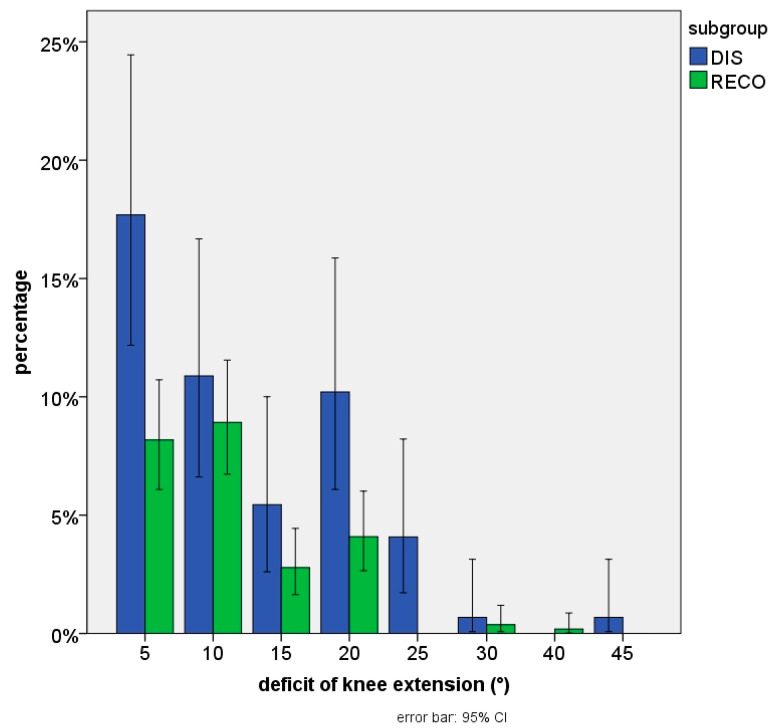
Proportion of patients with knee extension deficit on early follow-up.

Frequencies of major clinical findings are shown in Table 2. Active knee extension could not be fully demonstrated in 29.9% of all cases at the time of first follow-up. Although the extension deficit was mild (max. 5°) in most cases, we noticed this problem more frequently after DIS than after RECO (49.7% vs. 24.5%; *p* < 0.001). Following DIS, the problem not only seemed more frequent, but also more severe. Figure 3 shows those findings in detail. 

We advised 45 patients for revision (6.5%) during the first follow-up examination. This rate was significantly higher in DIS cases than in RECO cases (17.0% vs. 3.7%; *p* < 0.001). The proportion was also higher in females than in males (10.0% versus 4.6%; *p* = 0.006).

All in all, 63 patients (9.1%) underwent at least one revision operation. In addition, this proportion was significantly higher after DIS than after RECO (20.4% vs. 6.1%; *p* < 0.001). Female patients were significantly more frequently subject to operative revision (m/f: 7.1% versus 12.6; *p* = 0.015). An association between revision rate and patient age was not evident (Spearman’s ρ = 0.01; *p* = 0.8).

Following our strategy of early operative revision in cases of delayed regain of ROM, our most frequently applied revision procedure was arthroscopic adhesiolysis with anesthetic mobilization of the knee joint. A detailed overview of the procedures performed is given in Table 3.

## 4. Discussion

Recently the Dutch A.C.L. repair study group published DIS results from 155 patients treated in five centers and could not demonstrate non-inferiority of DIS versus RECO [46]. These results show the need for further research on both of these methods, which may contribute to improving operative techniques, patient selection, and decision making. Our similar study based on 690 patients focuses on early complications when evaluating the results of ACL surgery, either by DIS or RECO. Our 690-patient cohort underwent surgical treatment in our hospital between 2012 and 2022. In total, 147 patients were treated with DIS and 543 patients with RECO. 

As previously shown in several studies, the majority of patients are male [8,9,10]. However, the previously published gender difference regarding DIS was less pronounced in our collective. On average, female patients were slightly older than male patients, but in DIS cases, female patients tended to be younger, which correlates with previous studies showing that women suffer ACL ruptures at an earlier age than men [10,42,47].

Index operation was combined with medial meniscal surgery in 185 cases (resection: 14.9%; suture: 11.9%) and with lateral meniscal surgery in 147 cases (resection: 14.4%; suture: 7.0%). In the DIS group, meniscal suture, rather than resection, was the more frequently used method of treatment compared to RECO. This seems an interesting aspect worthy of further investigation. The most likely reason for this finding may be the shorter time interval between trauma and surgery in DIS patients. However, another hypothesis may be that injury mechanisms leading to proximal LCA rupture may be associated with meniscal injury more suitable for suture. Anyway, our findings are consistent with previous studies [20,34,35]. Other concomitant injuries accounted for only a small proportion and were treated according to current guidelines.

Postoperative follow-up visits at our clinic were recommended at 6 and 12 weeks. The mean duration until first follow-up was 53 days. We did not detect any cases of infection at the first follow-up. No significant differences were observed between DIS and RECO for most postoperative clinical findings, including effusion, swelling, rubor and circumferences. Anterior-posterior instability measured with a standard rolimeter also showed no significant differences. These findings indicate that both operative trauma burden and primary stability of RECO and DIS are comparable. This is remarkable because RECO—being associated with tendon harvesting and more extensive bone canal drilling—could be expected to perform worse than DIS on early follow-up. However, postoperative inflammation and healing processes, instead, seem equal. 

The fact that a significant proportion of patients (31.7% DIS and 41.4% RECO), despite explicit recommendation, did not receive any physiotherapy or rehabilitation at the time of the first follow-up is alarming. For both types of ACL surgery, early physiotherapeutic support, at least from the second week onwards, as recommended by the surgeons and the implant-manufacturers [44,45], is essential to counteract possible complications in the healing process [48]. Consistent physiotherapy is also important to avoid or correct permanent muscular asymmetries after cruciate ligament surgery. In a 2022 systematic review, it was shown that, in athletes after RECO, asymmetries in knee movements in the sagittal plane at first ground contact during the landing phase of a drop vertical jump are strong predictors of reinjury of the ACL. In addition, it was found that patients with muscular asymmetries of the lower extremity employed compensatory strategies for the hip, pelvis and trunk to compensate for imbalances.

Severe ROM restrictions (here defined as extension deficit of >10° or flexion deficit > 20°) were generally rare. However, this problem occurred significantly more frequently after DIS than after RECO (4.8% vs. 1.3%; *p* = 0.008). Mean knee flexion was more restricted after DIS (110°) compared to RECO (124°). The extension deficit of the knee joint was generally low, ranging from 1° to 5°. However, in comparison, it also occurred more frequently after DIS than after RECO, with a rate of 49.7% compared to 24.5%, respectively. Maybe this is partially explained by the significantly higher proportion of patients receiving simultaneous meniscal surgery in the DIS group (50.3% vs. 40.1%; *p* = 0.03). Other concomitant injuries, such as collateral ligament injury, accounted for only a small proportion and did not display significant differences. 

We advised 45 patients (6.5%) for revision surgery at the first follow-up examination, which is part of our strategy for arthrofibrosis prevention. The rate was significantly higher after DIS. In total, 63 patients (9.1%) underwent at least one revision surgery, with DIS patients being significantly more frequently affected than RECO patients (20.4% vs. 6.1%). Female patients were significantly more often subject to operative revision (m/f: 7.1% versus 12.6%). These high rates may be attributed to our consistent attitude to advice for aggressive early revision surgery whenever recovery of ROM is delayed. Accordingly, the most frequently performed procedure was arthroscopic adhesiolysis with anesthetic mobilization of the knee joint. The percentage of arthrofibrosis or restricted ROM after DIS, however, is consistent with previous studies, according to which the percentage is widely scattered between 18% and 33% [13,35,49,50,51]. In three cases it was necessary to change to an anterior cruciate ligament replacement after failed Ligamys stabilization, which is around double the implant failure rate of RECO. 

Evangelopulos et al. reported a combined complication rate of 78.8% that existed after DIS following midline ACL tears, of which 33.3% were due to extension deficits [49]. Recently the Dutch A.C.L. repair study group published the DIS results of 155 patients treated in five centers, which also could not demonstrate non-inferiority of DIS versus RECO [46]. They reported at least one complication in 25% of patients, comprising mainly arthrofibrosis, re-rupture and pain [46]. Most studies, including ours, have the limitation of short follow-up periods. Long-term results of our patient collective beyond the 12-week period are not known. In addition, some of the patients only showed up for one follow-up appointment. Furthermore, the examination results are influenced by the fact that a not insignificant proportion of patients had not yet received physiotherapy at the time of the follow-up appointments. The assessment of ROM deficits must therefore be viewed more critically. However, long-term study results to date following DIS tend to be ambivalent [13,34,42,52,53]. Moreover, a decision to use a particular surgical approach can neither be based solely on evidence of early complication rates nor on total complication rates. Complication rates per se do not evaluate the seriousness of the complications, which might be more or less easy to manage. Therefore, it is still important to establish a thorough indication respecting the individual patient’s goals, which might include the wish to preserve the “original” cruciate ligament and preserve potential autograft tendons. 

Strengths of our study are clearly the high patient numbers treated very consistently, regarding decision-making and operative technique, by only a small number of surgeons. Conversely, the single-center study design may also be regarded as a limitation. By focusing on early complication rates, aspects such as implant failure are probably underestimated. This limitation may distort a balanced comparison between DIS and RECO. 

Our findings are in line with the previous studies cited above, which also demonstrate increased complication rates after DIS compared to RECO. Technical modifications may also be a strategy to overcome this drawback. The combined complication rate could be reduced to 8.7% by applying a collagen I/III membrane to the surface of the ACL. This is in line with the findings of Murray in 2007 [31] and the successful treatment of an ACL wound with a collagen and platelet-rich plasma (PRP) hydrogel, which resulted in increased filling of the wound site with repair tissue that had increased profiles of growth factors and protein expression. Therefore, the standard use of a collagen membrane for Ligamys treatment should be considered in the future to reduce the risk of arthrofibrosis or overall limitation of knee joint motion.

## 5. Conclusions

Comparing early outcomes, ROM deficits were significantly more frequent after DIS than after ACL reconstruction. Between 30 and 40% of all patients did not receive any physiotherapy. For both DIS and RECO, early physiotherapeutic support, on average from the second week onwards, as recommended by most surgeons and the implant-manufacturers, is essential to counteract possible complications in the healing process [45,48]. Other reasons for the higher revision rate of DIS include the necessarily earlier timing of surgery for DIS, different drainage usage, a higher proportion of accompanying meniscal surgery, and differences in the surgical methods themselves. Usage of collagen membranes or platelet-rich-plasma may contribute to future improvements in the DIS method. 

A decision to use a particular surgical approach cannot be based solely on evidence of early complication rates. However, our findings are in line with several previous studies, which also demonstrated increased complication rates after DIS. Long-term study results to date following DIS tend to be ambivalent [13,34,42,52,53]. Still, it is important to establish a thorough indication respecting the individual patient’s goals, which may include preserving the “original” cruciate ligament and preserving potential autograft tendons. 

The need for immediate and regular physiotherapeutic co-treatment must be emphasized. In addition, we recommend routine follow-up examinations by the operating surgeon after 6 weeks, which can detect a significant proportion of patients who will profit from early arthroscopic revision (adhesiolysis or mobilization). 

## Figures and Tables

**Table 1:** Procedures combined with DIS and RECO (number, percentage in brackets).

Simultaneous Procedure	DIS	RECO	χ^2^
medial meniscus (resection)	17 (11.6%)	86 (15.8%)	n.s.
medial meniscus (suture)	17 (11.6%)	65 (12.0%)	
lateral meniscus (resection)	28 (19.0%)	71 (13.1%)	*p* = 0.002
lateral meniscus (suture)	18 (12.2%)	30 (5.5%)	
any simultaneuos meniscus surgery	74 (50.3%)	218 (40.1%)	*p* = 0.026
medical collateral ligament (suture)	3 (2.0%)	5 (0.9%)	n.s.
cartilage surgery (microfracuring)	2 (1.4%)	4 (0.7%)	n.s.
cartilage surgery (flake refixation)	1 (0.7%)	0 (0%)	n.s.

**Table 2:** Outcomes on early follow-up (ø 53 days) after ACL surgery.

	DIS	RECO	Significance
ROM restriction	4.8%	1.3%	*p* = 0.008
effusion	15.8%	20.7%	n.s.
swelling	42.5%	34.3%	n.s.
rubor	0.7%	0.2%	n.s.
mean knee flexion	110°	124°	*p* < 0.001
anteroposterior instability (rolimeter) compared to contralateral	−0.14 mm	+0.02 mm	n.s.
mean circumference thigh (20 cm above knee), compared to contralateral	−2.9 cm	−3.1 cm	n.s.
mean circumference thigh (10 cm above knee), compared to contralateral	−1.3 cm	−1.6 cm	n.s.
mean circumference knee, compared to contralateral	+0.8 cm	+0.8 cm	n.s.
mean circumference lower leg (15 cm below knee), compared to contralateral	−0.9 cm	−0.8 cm	n.s.

**Table 3:** Procedures performed for early revision surgery.

Revision Procedure	DIS	RECO	χ^2^
adhesiolysis/mobilization	24 (16.3%)	19 (3.5%)	*p* < 0.0001
revision for hemarthrosis (irrigation)	0 (0%)	1 (0.2%)	n.s.
revision for extracapsular hematoma	0 (0%)	3 (0.6%)	n.s.
revision for suspected infection (e.g., irrigation, synovectomy, local antibiotic treatment)	2 (1.4%)	2 (0.4%)	n.s.
revision graft	n.a.	6 (1.1%)	n.a.
conversion DIS → RECO	3 (2.0%)	n.a.	n.a.
resection of insufficient ACL	1 (0.7%)	2 (0.6%)	n.s.
Total	30 (20.4%)	33 (6.1%)	*p* < 0.0001
